# Amyloid Precursor Protein Is an Autonomous Growth Cone Adhesion Molecule Engaged in Contact Guidance

**DOI:** 10.1371/journal.pone.0064521

**Published:** 2013-05-14

**Authors:** Lucas J. Sosa, Jared Bergman, Adriana Estrada-Bernal, Thomas J. Glorioso, John M. Kittelson, Karl H. Pfenninger

**Affiliations:** 1 Department of Pediatrics and Colorado Intellectual and Developmental Disabilities Research Center, University of Colorado School of Medicine, Aurora, Colorado, United States of America; 2 Department of Biostatistics and Informatics, Colorado School of Public Health, University of Colorado Anschutz Medical Center, Aurora, Colorado, United States of America; Massachusetts General Hospital, United States of America

## Abstract

Amyloid precursor protein (APP), a transmembrane glycoprotein, is well known for its involvement in the pathogenesis of Alzheimer disease of the aging brain, but its normal function is unclear. APP is a prominent component of the adult as well as the developing brain. It is enriched in axonal growth cones (GCs) and has been implicated in cell adhesion and motility. We tested the hypothesis that APP is an extracellular matrix adhesion molecule in experiments that isolated the function of APP from that of well-established adhesion molecules. To this end we plated wild-type, APP-, or β1-integrin (Itgb1)- misexpressing mouse hippocampal neurons on matrices of either laminin, recombinant L1, or synthetic peptides binding specifically to Itgb1 s or APP. We measured GC adhesion, initial axonal outgrowth, and substrate preference on alternating matrix stripes and made the following observations: Substrates of APP-binding peptide alone sustain neurite outgrowth; APP dosage controls GC adhesion to laminin and APP-binding peptide as well as axonal outgrowth in Itgb1− independent manner; and APP directs GCs in contact guidance assays. It follows that APP is an independently operating cell adhesion molecule that affects the GC's phenotype on APP-binding matrices including laminin, and that it is likely to affect axon pathfinding *in vivo*.

## Introduction

Amyloid precursor protein (APP) and its two isoforms, APP-like proteins 1 and 2 (APLP1, 2), are transmembrane glycoproteins of the plasma membrane encoded by separate genes. They are subject to cleavage by three different proteases, which may release the Aβ peptide implicated in the pathogenesis of Alzheimer disease of the adult or aging brain [1], [2], [3]. Although APP has been known for a long time, its normal cellular functions and the role of its cleavage are poorly understood.

APP can bind to the extracellular matrix (ECM) components collagen I, laminin, spondin-1, reelin, glypican, and heparin, and it interacts or co-localizes with β1 integrin (Itgb1) and the actin-associated Ena/VASP-like protein [4], [5], [6], [7], [8], [9], [10], [11], [12]. While APP and Itgb1 s (Itga3b1 and Itga7b1) share the capacity to bind to laminin, the laminin binding sites are distinct [6], [13]. APP is abundant in growth cones (GCs) and has been implicated in cell migration and neurite outgrowth [11], [14], [15], [16], [17], [18], [19]. APP's enrichment in GCs [16] and the results of knock-out experiments [17], [20], [21] in particular suggest that it plays a critical role in the development of neuronal circuitry. The observations reviewed here are consistent with the hypothesis that APP may be an adhesion molecule or adhesion co-receptor of cells and GCs. However, direct evidence for this concept is not available in the literature.

Testing the hypothesis that APP is an axonal GC adhesion molecule faces two significant challenges, the large diversity of GC adhesion molecules [22], [23] and the difficulty of measuring GC adhesion. To address the former we used mono-specific synthetic matrices and neurons misexpressing APP and Itgb1 in order to isolate APP function. The matrices consisted of laminin-derived sequences that bind selectively to Itgb1 s or APP or of the external domain of the homophilic Ig superfamily adhesion molecule L1 (eL1). Several approaches have been used for assessing cell adhesion, including the application of force (fluid shear, centripetal force, atomic force microscopy, optical tweezers) and imaging by reflection interference contrast microscopy (RICM) and total internal reflection fluorescence (TIRF) microscopy. The application of fluid shear has been used to quantify GC adhesion [24], but the results, like those of the other displacement force measurements, depend greatly on the cell's or GC's cytoskeletal status and the direction of force application [25]. TIRF and RICM imaging can reveal adhesion sites characterized by the presence of adhesion-associated proteins [26] and the reduced space between cell and substrate (close adhesions [27]), respectively. Numerous reports link cell adhesions to close cell – matrix interactions as seen by RICM, and close adhesion area is linearly related to adhesive force over three orders of magnitude [28], [29], [30], [31], [32], [33]. Therefore, and because it does not perturb GCs, we used RICM to assess GC adhesions quantitatively. In addition, we measured GC detachment induced by competing, soluble substrate peptide, as a function of adhesion molecule expression and substrate type. Thus, the assays we used generated quantitative results on the specificity and extent of GC – matrix interactions.

To begin to understand what role APP plays in brain development we performed substrate choice assays to determine whether APP can participate in contact guidance mechanisms. Altogether, our findings establish in mouse hippocampal pyramidal neurons that APP is an autonomous axonal GC adhesion molecule involved in contact guidance.

## Results

### APP is Present in GC Adhesions on Laminin

**Figure 1:**
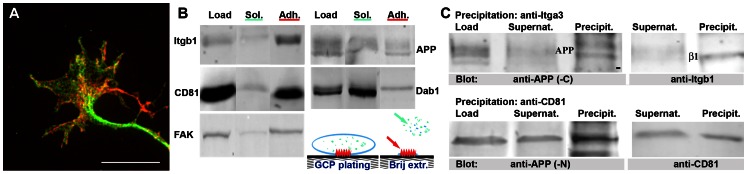
Presence of APP in GC adhesions. **A.** Immunofluorescence image of an axonal GC on laminin, fixed after 24 h *in vitro* and labeled with anti-APP (red) and anti-Itga3 (green). Note substantial overlap (yellow). Calibration, 10 µm. **B.** Isolation of GC adhesions on laminin. Rat GCPs plated on laminin were extracted with Brij98 to yield the unattached soluble fraction (see cartoon, green and blue; Sol). The remaining adherent structures (Adh, red) were recovered with SDS. The fractions were analyzed by western blot (equal fractional protein amounts loaded). Samples not extracted with Brij98 served as controls. The adhesion markers Itgb1, CD81 and FAK were enriched in the adherent fraction compared to its soluble counterpart, and significant amounts of APP and Dab1 (upper band) were detected. **C.** Co-immunoprecipitation of APP with Itga3 and CD81. The blots on the left show the enrichment of APP in the precipitates (N- or C-terminus-specific antibodies), while those on the right show precipitated Itgb1 (β1) and CD81.

Western blot analyses of GCs (GCPs) isolated by density gradient fractionation from newborn brain confirmed in mouse that APP was highly enriched in GCs (see [16]). This enrichment (about 7-fold relative to brain homogenate; not shown) was even greater than that of the well-established GC marker Gap43. Accordingly, a high level of APP immunofluorescence was characteristic of the axonal GCs of hippocampal pyramidal neurons in culture (Fig. 1A).

In order to assess whether APP was present in GC adhesions to laminin, we plated live GCPs isolated from fetal rat brain on laminin and extracted them with the mild detergent Brij98 (rat brain was used because of the sizable protein requirement). After extraction, the remaining adhesions were solubilized with SDS (cartoon in Fig. 1B). Figure 1B shows the enrichment, relative to the soluble fraction, of three markers of cell adhesions in the Brij98-resistant fraction, Itgb1, CD81, an Itg-associated tetraspanin, and focal adhesion kinase, FAK [34], [35], [36], [37]. In contrast, cell adhesion molecules *not* binding to laminin (such as NCAM and cadherin-2) were enriched, or found exclusively, in the Brij98-soluble fraction (data not shown). A large amount of APP was recovered in the adherent fraction, together with the upper band of the APP-binding protein Dab1 [18], [38], [39]. This result is consistent with the substantial overlap of APP and Itga3 immunoreactivities in axonal GCs on laminin, especially in the GC periphery and filopodia, where adhesions are concentrated (Fig. 1A; see also [15], [40], [41]). By co-immunoprecipitation we showed that APP forms Brij98-resistant complexes with Itga3b1 and the tetraspanin CD81 [34] in GCP adhesions to laminin (Fig. 1C; see also [11]). Together, these results extend previous reports and demonstrate the association of APP with laminin-bound GC adhesions.

### APP Misexpression Affects GC Structure and Function on Laminin

**Figure 2:**
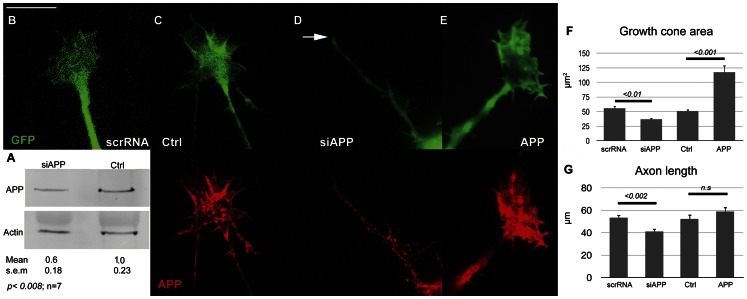
APP misexpression in wt mouse neurons on laminin. **A.** Western blot of hippocampal cultures treated with APP-targeted siRNA (siAPP) versus control siRNA. siAPP reduces the total APP level significantly (densitometric analysis: arbitrary values normalized to control; actin, loading control). **B–E.** Fluorescence images of neurons (24 h *in vitro*) transfected with GFP vector plus (left to right): scrambled siRNA (scrRNA), vehicle (Ctrl), siAPP, or APP vector, respectively. Top row, GFP fluorescence; bottom row, APP fluorescence. Arrow, fusiform GC. Scale bar 10 µm. **F, G.** GC area and axon length (means ± s.e.) in transfected neurons at 24 h *in vitro*. Horizontal bars, p values in two-sample t tests. n.s., not significant.

If APP is involved in GC adhesion to laminin then APP gain and loss of function should affect GC spreading and advance on this substrate. APP-targeted siRNA (siAPP) significantly reduced total APP protein in hippocampal cultures (Fig. 2A) and APP immunofluorescence in axonal GCs (Fig. 2D). Average GC pixel intensity was decreased by 42±5% (mean ± s.e., p<0.005, n = 15). In contrast to the control siRNA, siAPP reduced GC spread on laminin (Fig. 2D) to about 35% of control. APP overexpression, however, more than doubled GC size (Fig. 2E, F; note increased APP fluorescence). APP misexpression also affected axonal growth (axon length after the first 24 h in culture). While lengths of APP-overexpressing axons were not significantly different from controls, APP-knockdown significantly shortened them by about 25% (Fig. 2G).

**Figure 3:**
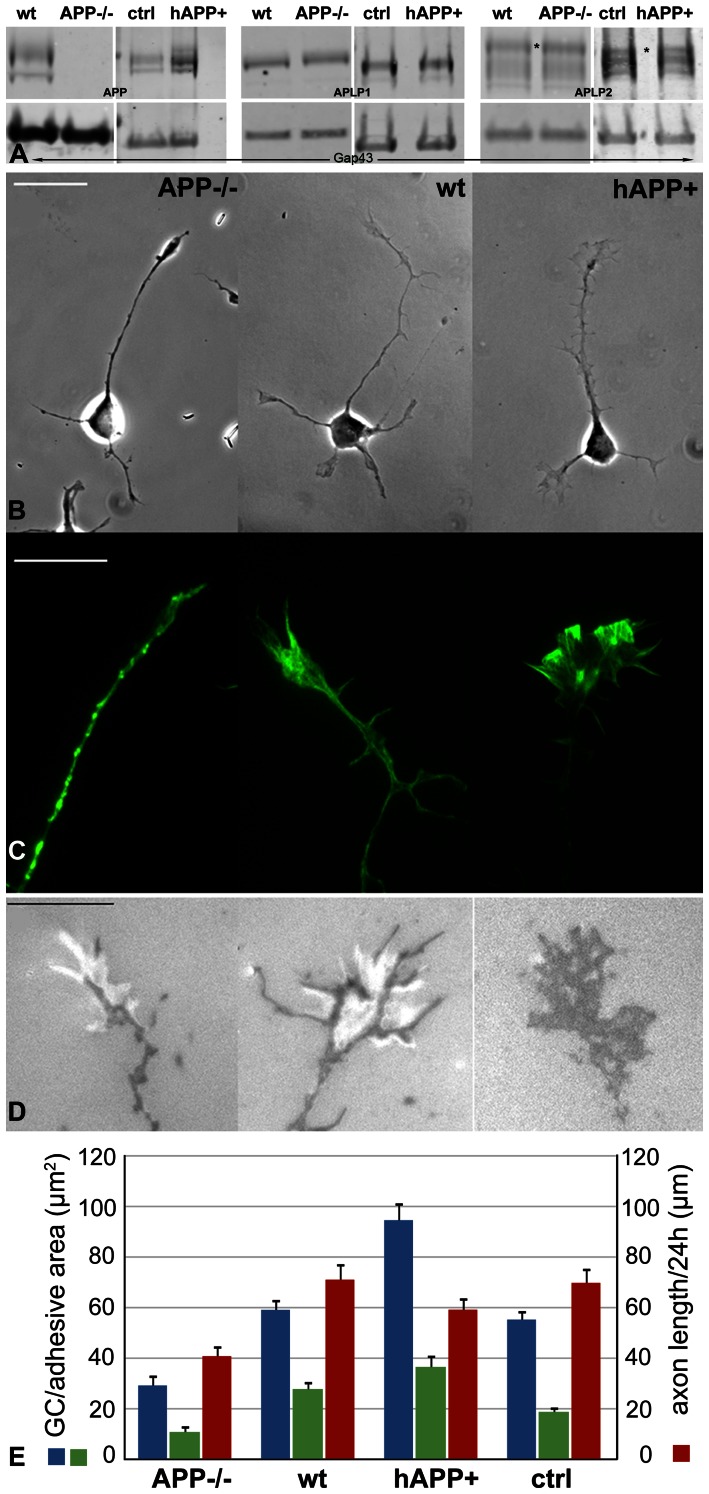
GC size, adhesion and advance of APP mutant neurons on laminin. **A.** Western blots of GCPs isolated from newborn brain of wt and mutant mice. Blots were probed for APP (M_r_ ∼100 kDa), APLP1 (M_r_  = 72 kDa), and APLP2 (M_r_  = 87 kDa, asterisk) and for Gap43 as loading control. APLP1 and APLP2 levels were the same in wt and mutant mice. **B.** Phase contrast images of wt and mutant neurons and (**C**) TIRF images of their GCs labeled with fluorescent phalloidin. **D.** RICM images of live GCs showing close adhesions (dark) and wide contacts (bright). hAPP+ control GCs (ctrl; non-transgenic siblings) were the same as wt and, therefore, are not shown. Scale bars, 20 µm (**B**) and 10 µm (**C, D**). **E.** GC area (blue), cumulative adhesive area (green) and axon length (red) at 24 h *in vitro* for the indicated mouse strains (means ± s.e.). For statistical analysis, see Tables 1 and 2.

Similar experiments were performed with hippocampal pyramidal neurons from an APP knock-out mouse (APP−/−; [42]) and a transgenic mouse expressing a copy of wt human APP in addition to the mouse alleles (hAPP+; [43]). We isolated GCPs from the brains of wt and mutant mice and analyzed Western blots for levels of APP, APLP1 and APLP2 (Fig. 3A). Gap43 immunoreactivity was used as loading control. APP protein was increased (1.9-fold) in hAPP+ but not detectable in APP−/− GCPs, and we did not detect compensatory changes in APLP1 or 2 levels. On laminin, axonal GC sizes changed with APP expression levels as described for the transfected neurons (Fig. 3B, C). Live GCs were examined by RICM, which reveals close adhesions as dark and wider contacts as white areas (Fig. 3D; [27], [32]). Cumulative area of close adhesion, total GC area, and axon length after 24 h *in vitro* were analyzed quantitatively and statistically (Fig. 3E and Tables 1, 2). Together with total GC size, close adhesion areas were significantly reduced in APP−/− GCs compared to wt controls, whereas they were greatly increased in hAPP+ GCs relative to their controls (non-transgenic littermates). The numbers of GC filopodia were reduced in APP−/− GCs vs. wt (2.1±0.3 vs. 3.3±0.3 filopodia/GC, respectively; p≤0.008, n = ≥14) but substantially increased in hAPP+ GCs (4.4±0.6 filopodia/GC; p≤0.013; n≥19). Initial outgrowth of the mutant neurons (at 24 h in culture) paralleled that of the transfected neurons, with the hAPP+ axons not significantly different, but the lengths of the APP−/− axons significantly reduced. Because the neurons of non-transgenic littermate controls of hAPP+ mice were not distinguishable from wt we compared the hAPP+ mutants to wt in the subsequent experiments.

**Table 1:** GCs on Laminin, Numerical Data.

Neuron	GC Size (µm^2^)			Close Adhesions (µm^2^)			Axon Length (µm)		
	N	Mean	SD	N	Mean	SD	N	Mean	SD
**Wt**	12	59.13	14.51	21	27.77	14.84	25	71.06	31.4
**APP−/−**	11	29.2	13.97	21	10.84	12.18	20	40.78	18.9
**hAPP+**	19	94.56	28.48	20	36.53	21.08	20	59.19	19.82
**Ctrl**	23	55.26	16.97	18	18.79	9.02	23	69.77	27.74

**Table 2:** GCs on Laminin, Statistical Analysis.

Comparison		Av. Diff.	S.E.	95% Confid. Interv.	P-Value
**GC Size (µm^2^)**	wt vs. APP−/−	−29.92	5.94	(−42.28, −17.57)	0.0001*
	ctrl vs. hAPP+	39.29	7.43	(24.08, 54.51)	<0.0001*
**Close Adh. Area (µm^2^)**	wt vs. APP−/−	−16.93	4.19	(−25.41, −8.45)	0.0002*
	ctrl vs. hAPP+	17.74	5.17	(7.11, 28.36)	0.0020*
**Axon Length (µm)**	wt vs. APP−/−	−30.27	7.57	(−45.57, −14.98)	0.0003*
	ctrl vs. hAPP+	−10.58	7.29	(−25.31, 4.15)	0.1545

* ) Significant.

### Neurons on Mono-Specific Matrices

To test the adhesive function of APP in GCs and compare it to that of well-established adhesion molecules, such as integrins (Itga3b1 and Itga7b1 are present in these GCs) and L1, we prepared culture matrices of laminin peptides that bind selectively either the integrins (integrin-binding peptide, Ibp [13]) or APP (APP-binding peptide, Abp [6]), and of recombinant eL1, a fusion protein with the Fc region of IgG (generous gift of M. Grumet, Rutgers [44]). We analyzed GC adhesion and axonal outgrowth of wt, APP−/−, hAPP+ and Itgb1− neurons on the mono-specific matrices. The Itgb1− neurons were generated by transfection of wt neurons with Itgb1− targeted siRNA (siItgb1) and a GFP-encoding plasmid for identification.

**Figure 4:**
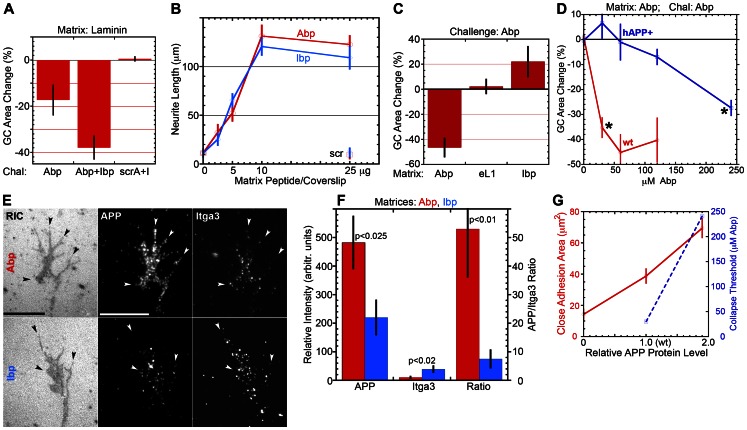
GC adhesion to peptide matrices and adhesion assessment in competition experiments with soluble peptides. **A.** Wt mouse neurons were grown on laminin and challenged for 10 min with approx. 37 µM each of Abp, Abp plus Ibp or scrambled Abp plus scrambled Ibp (scrA+I). Peptide-induced GC detachment is shown as area reduction (mean % change ± s.e., relative to area before challenge; n≥9). Student's t tests were: for Abp vs. Abp+Ibp and for Abp vs. scr A+I, <0.05; for Abp+Ibp vs. scrA+I, <0.0001. **B.** Neurite length (mean ± s.e.) at 24 h in culture, as a function of peptide deposition. Matrix peptide/coverslip, amount used for coating. scr, data points for scrambled Abp and Ibp. **C.** Wt mouse neurons were grown on Abp, eL1 or Ibp and challenged with 75 µM soluble Abp for 10 min. Graph shows Abp-induced change in GC area (mean % change ± s.e.). GC collapse (p<0.001; n ≥4) was observed only on Abp (Ibp was not significantly different from eL1). **D.** Comparison of wt and hAPP+ GCs in collapse assays on Abp (10 min challenge). GC area change (mean % ± s.e.) is shown as a function of Abp concentration applied. Asterisks mark the lowest Abp concentrations triggering significant collapse (threshold p = 0.05; actual significance, p≤3.0E-5). **E.** RICM and TIRF microscopy of wt GCs (grown for 24 h on Abp or Ibp) fixed, permeabilized and labeled with anti-APP and anti-Itga3. Close adhesions are co-extensive with clustering of APP and Itga3 on Abp and Ibp, respectively (arrow heads). Bars, 10 µm. **F.** Fluorescence intensity (mean ± s.e., n = 6) of anti-APP and anti-Itga3, and ratio of these intensities in GCs on Abp (red) and Ibp (blue). Antigen clustering is substrate-specific. Labeling intensities of APP and Itga3 cannot be compared because the antibody affinities are unknown. **G.** GC close adhesion area (red line; from Table 3) and collapse threshold (dashed blue line; from D) plotted relative to APP protein level (normalized to wt; see Fig. 3).

The peptides were tested in wt GC collapse experiments on laminin (Fig. 4A). Total GC areas were measured prior to challenge and 10 minutes thereafter. Upon challenge with Abp alone we observed 17% GC detachment (reduction in area; p<0.05 compared to scrambled peptides), and this was doubled when both Abp and Ibp were applied (p<0.05; n≥9). However, the scrambled sequences did not affect GC spread at all (Abp + Ibp vs. scrambled peptides, p≤0.0001). This result indicated that Abp and Ibp were bioactive and that GC – laminin adhesion included an Abp-sensitive component. Next, we measured wt neurite outgrowth, over the first 24 h *in vitro,* as a function of peptide concentration used for matrix preparation. Figure 4B shows that, in the absence of Abp or Ibp or in the presence of scrambled peptide, there was virtually no outgrowth. Increasing peptide concentration, however, enhanced neurite outgrowth until it plateaued at >10 µg per coverslip for both peptides. Thus, each peptide sustained neurite outgrowth by itself, in a concentration-dependent manner.

We performed additional peptide competition experiments (i) to determine specificity of the GC – matrix interaction and (ii) to obtain an independent measure of adhesive strength. For the former, wt neurons were grown for 24 h on the 3 synthetic matrices and challenged with 75 µM soluble Abp. We observed statistically significant GC collapse on Abp, but not on Ibp or eL1 (Fig. 4C). Successful competition with binding to Abp indicated that, on this matrix, GC adhesion was indeed mediated by APP binding. In a second set of experiments we compared collapse of wt and hAPP+ GCs on Abp as a function of the concentration of soluble Abp (Fig. 4D). The threshold Abp concentration for GC collapse of hAPP+ neurons was about 8 times greater than that of wt neurons. The increased Abp resistance of GCs overexpressing APP (1.9 x wt level; Fig. 4G) indicates greater adhesive strength.

Having demonstrated formation of biologically meaningful GC – matrix adhesions via APP we examined them by RICM and TIRF microscopy. Wt neurons were fixed minimally, permeabilized with mild detergent and labeled with anti-APP and anti-Itga3. RICM of fixed specimens was inferior to that of live GCs, but it revealed adhesions on both peptides, with APP and Itga3 clustered in the close adhesions on Abp and Ibp, respectively (Fig. 4E). Quantitative analyses of fluorescence intensity (TIRF) showed that each adhesion molecule was at least twice as abundant when the membrane adhered to its ligand vs. the other peptide (Fig. 4F), and the APP/Itga3 ratio was about 7-fold higher on Abp than on Ibp (p<0.01; n = 6). Therefore, the imaged close contact areas of GCs were physiologic adhesions.

**Figure 5:**
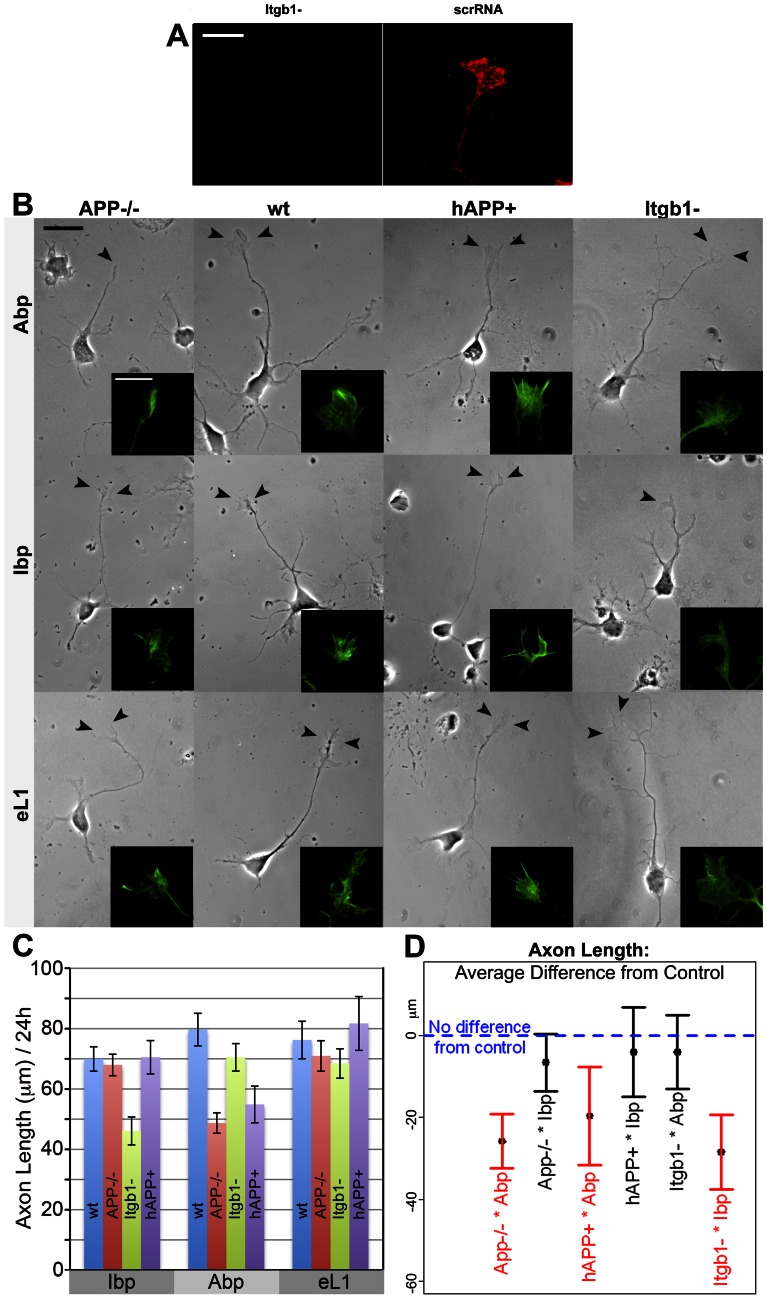
Axon length of APP mutant and Itgb1− deficient mouse neurons on monospecific substrates. **A.** Knock-down of Itgb1 protein in wt GC by siItgb1 (control, scrRNA). Itgb1 fluorescence (red) was not detectable at 24 h *in vitro*. **B.** APP mutant and Itgb1− neurons on mono-specific matrices for 24 h (phase contrast). Scale 20 µm. Arrow heads point at the axonal GCs. Inserts show these GCs at higher magnification (bar 10 µm) after phalloidin labeling (APP−/−, wt, hAPP+) or to reveal the GFP transfection marker (Itgb1−). **C.** Axon lengths for different neurons on the three growth substrates (bottom labels) after 24 h *in vitro* (means ± s.e.). **D.** Average difference in outgrowth and associated 95% confidence interval for each neuron*matrix combination relative to the control group. Negative values indicate average growth below that of the control group. The only significantly different combinations (red) are APP−/− and hAPP+ on Abp, and Itgb1− on Ibp.

**Figure 6:**
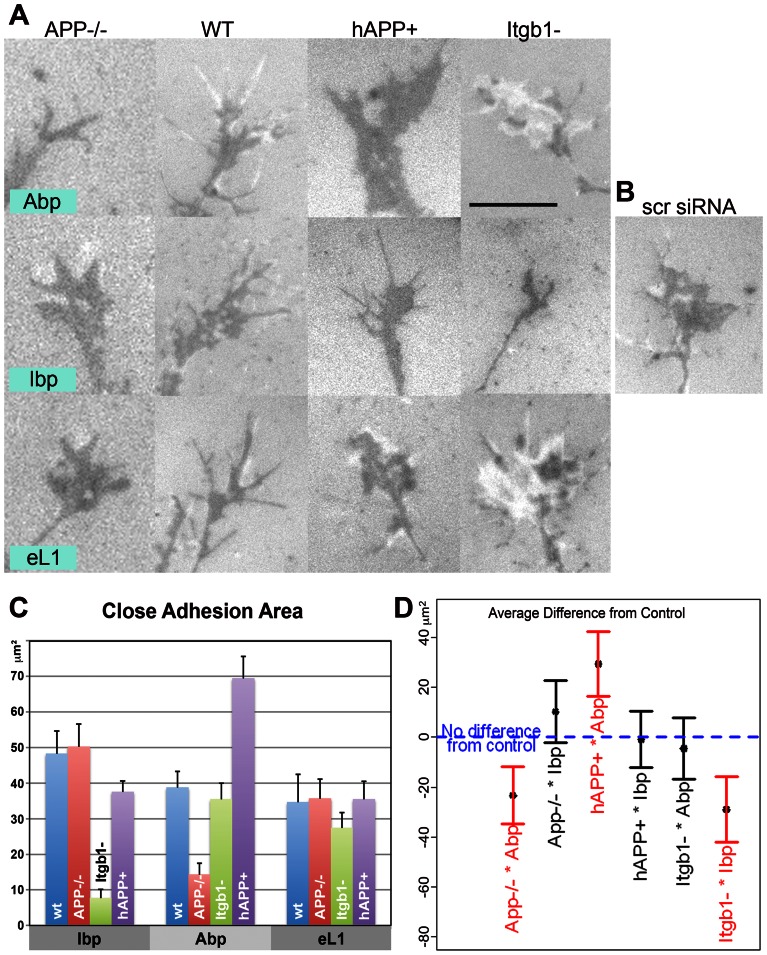
GC adhesion of APP mutant and Itgb1− deficient mouse neurons to mono-specific matrices. **A.** RICM of live GCs of mutant neurons (columns) on different matrices (rows). Dark areas indicate close adhesions, bright areas are wider contacts. Scale 10 µm. Note different adhesive areas of APP−/− and hAPP+ GCs on Abp, and of the Itgb1− GC on Ibp. **B.** RICM image of live GC of a neuron transfected with scrambled siRNA on Ibp [control for adjacent image (Itgb1− on Ibp)]. **C.** Cumulative close adhesion areas of GCs in different neuron*matrix combinations (means ± s.e.). **D.** Average difference of close adhesion areas from control group and the associated 95% confidence intervals for the tested neuron*matrix combinations. Negative values indicate reduced and positive values indicate increased areas. Only the values for APP−/− and hAPP+ neurons on Abp, and for Itgb1− neurons on Ibp are significantly different (red).

Comparison of the 4 different neuron types on the 3 matrices is illustrated in Figures 5 and 6. To evaluate the quantitative data we used a two-stage statistical analysis to control the risk of Type I errors. Because the behavior of wt neurons should not be (and, indeed, was not) altered on the three matrices (Table 3), and because the mutations of the other neurons should not (and did not) affect behavior on the eL1 matrix, these six values constituted the control group. The remaining six combinations were each compared separately to the average of the control-group means using a linear contrast in the means-model described in Methods. The cut-off p-value used to define statistical significance was determined by Monte Carlo simulation to control the false-positive risk (Type I statistical error) under the null hypothesis (Tables 4, 5).

**Table 3:** GCs on Synthetic Matrices. Numerical Data: Mean (SD, N).

Parameter	Matrix	Neuron Type			
		Wt	APP−/−	hAPP+	Itgb1−
**Axon Length (µm)**	Abp	79.69 (26.94, 26)	48.72 (20.23, 73)	54.87 (13.98, 17)	70.5 (25.74, 33)
	Ibp	69.94 (18.52, 22)	67.97 (18.59, 63)	70.52 (18.89, 21)	46.09 (16.22, 32)
	eL1	76.22 (29.77, 24)	70.95 (29.82, 36)	81.71 (38.88, 20)	68.43 (27.11, 32)
**Close Adh. Area (µm^2^)**	Abp	45.16 (40.55, 26)	16.77 (20.33, 28)	69.5 (26.44, 20)	35.53 (21.85, 23)
	Ibp	48.28 (25.41, 17)	50.34 (29.57, 23)	39.16 (17.34, 29)	11.1 (10.0, 20)
	eL1	45.1 (50.49, 16)	39.06 (28.02, 22)	35.54 (18.04, 14)	27.38 (16.22, 15)

**Table 4:** GCs on Synthetic Matrices. ANOVA Values.

Parameter		Degr. Freed.	Sum. Sq.	Mean Sq.	F-Value	Pr(>F)
**Axon Length (µm)**	Matrix	2	12200.66	6100.33	10.79	0.0001*
	Neuron	3	11893.04	3964.35	7.01	0.0001*
	Matrix*Neuron	6	27035.6	4505.93	7.97	<0.0001*
	Residuals	387	218862.03	565.53		
**Close Adh. Area (µm^2^)**	Matrix	2	336.12	168.06	0.23	0.7973
	Neuron	3	21166.88	7055.63	9.52	<0.0001*
	Matrix*Neuron	6	34840.98	5806.83	7.84	<0.0001*
	Residuals	241	178589.02	741.03		

* ) Significant.

**Table 5:** GCs on Synthetic Matrices – Statistical Analysis.

Combination (neuron on matrix)		Mean Diff. (Control-Comb.)	S.E.	95% Confidence Interval	P-Value
**A.** Axon Length (µm) – Significance Level = 0.0091					
APP−/− on Abp		25.77	3.38	(19.12, 32.42)	<0.0001*
APP−/− on Ibp		6.52	3.56	(−0.48, 13.51)	0.0679
hAPP+ on Abp		19.62	6.08	(7.67, 31.57)	0.0014*
hAPP+ on Ibp		3.97	5.53	(−6.91, 14.84)	0.474
Itgb1− on Abp		3.99	4.56	(−4.98, 12.96)	0.3827
Itgb1− on Ibp		28.4	4.62	(19.32, 37.49)	<0.0001*
**B.** Close Adhesion Area (µm^2^) – Significance Level = 0.0088					
APP−/− on Abp	23.32		5.79	(11.91, 34.72)	<0.0001*
APP−/− on Ibp	−10.26		6.27	(−22.6, 2.09)	0.1031
hAPP+ on Abp	−29.41		6.64	(−42.5, −16.33)	<0.0001*
hAPP+ on Ibp	0.92		5.71	(−10.33, 12.17)	0.8717
Itgb1− on Abp	4.56		6.27	(−7.79, 16.9)	0.4677
Itgb1− on Ibp	28.98		6.64	(15.9, 42.07)	<0.0001*

*Significance Level* is the cut-off used to define statistical significance in order to control risk of type I error.

* ) indicates p-values smaller than *Significance Level.*

Wt neurons grew uniformly well on all 3 matrices, with equal initial outgrowth (Fig. 5B, C; Table 3). Close adhesion areas of axonal GCs did not significantly differ either (Fig. 6A–C; Table 3). APP−/− axons grew equally well, with the same GC parameters, on Ibp and eL1. On Abp, however, initial outgrowth was reduced to 61% of wt. APP−/− GCs were fusiform, and their adhesive area was much smaller (37% of wt) (Fig. 5B–D; Table 5; Fig. 6A, C, D). Itgb1− neurons, identified by their GFP label (Fig. 5B, inserts, right column) and shown to exhibit reduced Itgb1 immunoreactivity (Fig. 5A), grew normally on the Abp and eL1 matrices and were indistinguishable from wt neurons or control neurons transfected with scrambled siRNA (Figs. 5B–D, 6; Table 5). On Ibp, however, the Itgb1− axonal GCs were adhesion-deficient and advanced at a significantly reduced rate (66.5% of control; Figs. 5B–D, 6; Tables 3, 5). The hAPP+ axons grew well on the Ibp and eL1 matrices (Tables 3, 5; Figs. 5B–D and 6). On Abp, however, GCs advanced significantly less in the first 24 h compared to the control group (Figs. 5B–D; Table 5A). These very large GCs had close adhesion areas increased to 154% of wt (Figs. 5, 6; Tables 3, 5B).

In summary, GC adhesion and initial outgrowth for wt neurons on the 3 matrices and for wt and mutant neurons on eL1 were essentially the same. Distinctive close adhesion values were seen only for Itgb1− GCs on Ibp and for APP-misexpressing neurons on Abp (Fig. 6D). In the latter, APP levels in GCs, determined by Western blot, ranged from 0.0 for APP−/− to 1.9 for hAPP+ when normalized to 1.0 for wt (arbitrary units). Plotted against this scale, close adhesion area on Abp increased approximately linearly (Fig. 4G). Initial outgrowth was reduced relative to control for *both* APP−/− and hAPP+ neurons but unchanged on Ibp and eL1 (Tables 3, 5A; Fig. 5D).

### APP Influences Axonal Contact Guidance

**Figure 7:**
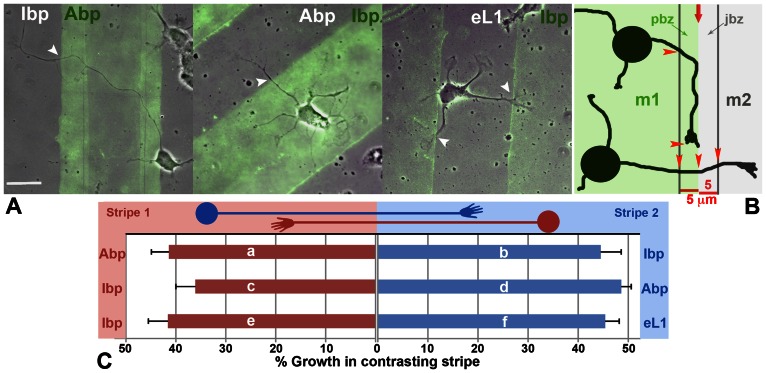
Substrate choice control assays. **A.** Wt neurons grown for 24 h on alternating lanes of the indicated synthetic substrates. The stripe deposited first was quenched with fluorescent BSA (green; superimposed TIRF and phase contrast images). White arrowheads point at neurite crossings from one substrate to the other. The neurites grew without substrate preference. Scale 20 µm. **B.** Cartoon to explain the border zone analysis. m1 and m2, matrix 1 and 2. The red arrow marks the border between them. Two bands, 5 µm wide, define the proximal (pbz) and juxtaposed (jbz) border zones. Arrowheads indicate measured axonal segments. **C.** Percent growth in juxtaposed stripe (border zone analysis). The frame of the bar graph identifies the substrate pairings, with “stripe1” and “stripe 2” deposited first and second, respectively. The cartoons of the neurons designate the position of their perikarya and growth directions. The bar (mean ± s.e.) in the same color as the neuron indicates % growth in the juxtaposed stripe. Letters a to f refer to the numerical data and statistics in Tables 6 and 7.

Our data indicate that APP is an axonal GC adhesion molecule. To determine whether it may participate in pathfinding we used the stripe assays pioneered by Bonhoeffer and collaborators [45], [46], [47] and plated wt or mutant neurons on alternating lanes of two of the synthetic substrates. Figure 7A shows wt neurons on 3 of the possible permutations (24 hr *in vitro*). Wt axons appeared to grow randomly across the different stripes, without preference for any of the substrates, and regardless of whether a substrate was deposited first (quenched with fluorescent BSA) or second. The latter was an important consideration because the second matrix was applied across the initial stripes and, thus, could alter their properties. We analyzed these control experiments with 3 quantitative methods.

*a) Relative neurite lengths on different stripes, using stereology.* The data obtained for Abp vs. Ibp stripes (the matrix listed first always is the one deposited first) showed that neurites originating from the Abp (first) stripe were just as likely to cross onto, and grow on, the juxtaposed matrix as those from the Ibp (second) stripe (Table 6). These values were relatively low because many neurites grew at some distance from the interface and, therefore, did not encounter the juxtaposed matrix.

**Table 6:** Abp versus Ibp Controls.

Assessment	1^st^/2^nd^ Matrix	Growth Direction	Ref.	% cross-over (mean ± s.e.)
**Stereology** 1	Abp/Ibp	Ibp to Abp	-	29.3±4.8
	Ibp/Abp	Abp to Ibp	-	30.7±4.7
**Binary choice** 2	Abp/Ibp	Ibp to Abp	-	43.8±7.6
	Ibp/Abp	Ibp to Abp	-	50.4±10.5
	Abp/Ibp	Abp to Ibp	-	59.5±6.7
	Ibp/Abp	Abp to Ibp	-	49.8±6.5
**Border zone** 3	Abp/Ibp	Ibp to Abp	a	41.4±3.49
	Ibp/Abp	Ibp to Abp	d	48.56±2.03
	Abp/Ibp	Abp to Ibp	b	44.1±4.29
	Ibp/Abp	Abp to Ibp	c	35.68±4.14

1 ) Relative amount of outgrowth (% of cumulative neurite length) on contrasting lane. n = 23.

2 ) Relative number of growth cones that had entered the proximal 5-µm border zone and crossed into the juxtaposed stripe, as % of total number of growth cones observed in the proximal border zone. n = 3.

3 ) Neurite growth in distal border zone (relative to that in proximal and distal border zones combined). n≥17.

Ref., Reference to Figure 7.

*b) The binary choice analysis* determined the percentage of GCs crossing into the adjacent lane (by at least 5 µm) once they entered a zone 5 µm wide and proximal to the border between two stripes. The 5 µm width was selected because GC filopodia could spread beyond it and detect the adjacent, potentially preferred substrate. This assay seemed to indicate GC preference for the second substrate in Abp vs. Ibp and Ibp vs. Abp juxtapositions, but the numbers were not significantly different (Table 6).

*c) The border zone analysis* determined the lengths of a neurite within two 5 µm-wide zones on either side of the interface between two lanes (Fig. 7B). The rationale for this assay was that once, and as long as, a GC was within a border zone it could detect the presence of the juxtaposed matrix and, thus, choose the preferred substrate. Persistent growth on one side of the border thus indicated preference for that matrix. Numbers denote lengths of neurite outgrowth within the *border zone juxtaposed to the neurite*'*s lane of origin*, expressed as % of total neurite length within the two border zones. Therefore, values near 50% indicate no substrate preference, whereas values well below 40% demonstrate reduced crossing onto the juxtaposed matrix and, thus, preference for the stripe of origin. These measurements (Fig. 7B; Tables 6, 7), like those described for the binary choice analysis (Table 6), suggested a slight preference for the stripe deposited second in both Abp-to-Ibp and Ibp-to-Apb assays, but the numbers differed significantly only when Ibp was deposited first (Fig. 7C; Table 7A). In Ibp vs. eL1 pairings there was no growth preference in either direction (Table 7A). Overall, these control studies indicated that wt axons did not prefer any one of the 3 synthetic matrices. However, because of the slight preference for the matrix deposited second we laid down permissive or preferred substrates (as concluded from initial experiments) first and candidate non-permissive or non-preferred matrices second in the experiments described below. This excluded any possibility of bias introduced by the order in which the substrate lanes were generated.

**Table 7:** Haptotactic Assay Controls, wt Neurons.

1^st^/2^nd^ Matrix	Direction	Ref.	Mean % cross-over (SD, N)	Mean Diff.	S.E.	95% Confid. Interval	P-Value^+^
**A.** Growth Direction							
Abp/Ibp	Ibp to Abp	a	41.4 (16.75, 24)				
	Abp to Ibp	b	44.1 (18.69, 20)	2.7	5.39	(−8.22, 13.63)	0.6195
Ibp/Abp	Abp to Ibp	c	35.68 (17.06, 18)				
	Ibp to Abp	d	48.56 (8.11, 17)	12.88	4.65	(3.65, 22.11)	0.0082*
Ibp/eL1	eL1 to Ibp	e	41.65 (17.75, 22)				
	Ibp to eL1	f	45.4 (13.23, 24)	3.75	4.65	(−5.66, 13.15)	0.425
**B.** Order of Matrix Deposition							
Abp/Ibp	Ibp to Abp	a	41.4 (16.75, 24)				
Ibp/Abp		d	48.56 (8.11, 17)	7.16	3.94	(−0.85, 15.17)	0.078
Ibp/Abp	Abp to Ibp	c	35.68 (17.06, 18)				
Abp/Ibp		b	44.1 (18.69, 20)	8.42	5.8	(−3.34, 20.18)	0.1553

Ref., Reference to Figure 7.

+) Significance Level  = 0.05.

* ) Significant.

**Figure 8:**
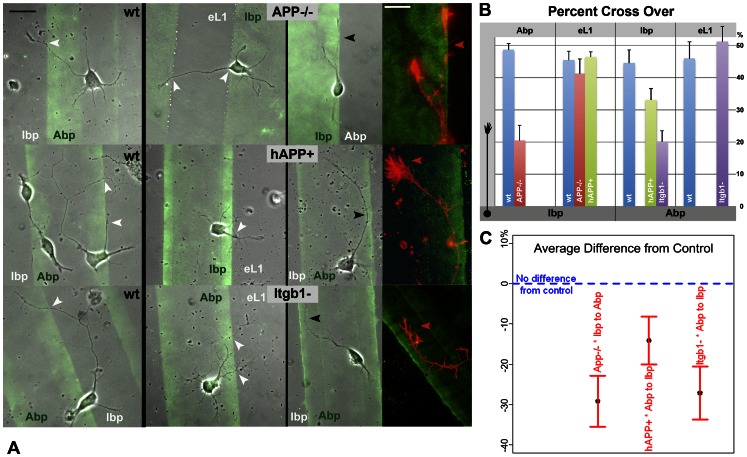
Substrate choice assays of wt versus mutant GCs. Neurons were grown for 24 h on different matrix pairings. The stripe deposited first is green fluorescent. **A.** Superimposed phase contrast and TIRF images of wt (left column) and mutant neurons (center and right columns). Scale 20 µm. White arrowheads mark neurite crossings from one matrix to another. Axonal GCs with a substrate preference, i.e., avoiding the juxtaposed matrix (black arrowheads), are shown in the right column, together with fluorescence images of phalloidin label (red) of the same structures at higher magnification (bar 10 µm). Note the very large hAPP+ GC on Abp. **B.** Border zone analysis to indicate growth on juxtaposed matrix (percent cross-over) for wt and mutant neurons on different substrate pairings. The growth direction is shown by the neuron cartoon on the left, with the perikaryon on the matrices indicated below and the juxtaposed substrates listed on top. Most combinations (including all experiments with wt neurons) exhibit no growth preference. **C.** Average difference from control group and associated 95% confidence interval for the three significantly different combinations of neuron type*substrate pairings/growth directions. Negative values indicate preference for the substrate of origin.

Figure 8A shows wt and mutant neurons (Itgb1− were wt transfected with siItgb1) on 3 different substrate pairings (the right column includes fluorescence images of phalloidin label of the same axons shown in phase contrast, at higher magnification). Border zone analysis generated the quantitative data in Figure 8B (bottom and top indicate the substrate in the stripe of axon origin and that in the contrasting lane, respectively). ANOVA analyses were performed as described (Tables 8–10), with the wt neurons on all matrix pairings and the mutant neurons on permissive substrate-eL1 juxtapositions serving as the control group. Figure 8C shows the average differences from control with the 95% confidence intervals. APP−/− neurites, which behaved like wt in the Ibp vs. eL1 experiment, avoided Abp when compared to the combination control group (Fig. 8A, top of right column). Analogously, Itgb1− neurites, which grew equally well on Abp and eL1, crossed significantly less from Abp onto Ibp compared to control (Fig. 8A, bottom of right column). Remarkably, hAPP+ neurites, which grew like wt in the Ibp-eL1 juxtaposition, significantly preferred Abp in the Abp vs. Ibp experiment (Fig. 8A, center of right column). It follows from these experiments that (i) Itgb1 knock-down interfered with axon crossing onto Ibp only, and that (ii) APP misexpression affected the neurite choice between Abp and the other substrates in negative or positive direction.

**Table 8:** Haptotactic Assays. Numerical Data: Mean % Cross-Over (SD, N).

Comparison Group	Neuron Type			
	Wt	APP−/−	hAPP+	Itgb1−
**Abp – Ibp**	46.15 (14.79, 37)	18.04 (18.21, 29)	33.09 (18.2, 34)	20.05 (16.84, 26)
**Ibp – eL1**	45.4 (13.23, 24)	46.21 (13.93, 31)	46.36 (6.31, 17)	–
**Abp – eL1**	47.88 (13.74, 20)	–	–	51.19 (19.53, 17)

**Table 9:** Haptotactic Assays. ANOVA Values for % Cross-Over.

	Degr. Freed.	Sum. Sq.	Mean Sq.	F-Value	Pr(>F)
**Matrix**	2	15945.24	7972.62	32.55	<0.0001*
**Neuron**	3	7197.04	2399.01	9.8	<0.0001*
**Matrix** * **Neuron**	3	9533.79	3177.93	12.98	<0.0001*
**Residuals**	226	55349.24	244.91		

* ) Significant.

**Table 10:** Haptotactic Assays. Statistical Analysis.

Combination		Mean Diff. (Contr.-Comb.)	S.E.	95% Confidence Interval	P-Value+
Neuron	Matrices/Direction				
APP−/−	Ibp to Abp	29.16	3.21	(22.84, 35.47)	<0.0001*
hAPP+	Abp to Ibp	14.11	3.01	(8.19, 20.03)	<0.0001*
Itgb1−	Abp to Ibp	27.14	3.35	(20.54, 33.75)	<0.0001*

+) Significance Level  = 0.0172.

* ) Significant.

## Discussion

Previous observations that APP can bind to ECM proteins, that it interacts or co-localizes with Itgb1 and the actin-associated Ena/VASP-like protein, and that it affects cell migration and neurite outgrowth have long suggested that it is involved in cell adhesion to the ECM (for references, see Introduction). Other data indicate APP and/or APLPs can form homo- and hetero-complexes, in trans, that promote intercellular adhesion ([48]; see also [49]). However, the diversity of adhesion molecules on cell surfaces and the multiplicity of cellular binding sites in ECM preparations (including laminin) make it difficult to test the adhesion hypothesis [23]. Therefore, we used mono-specific substrates in combination with neurons misexpressing APP. Most neurons were from mutant mice, in which APP protein levels were consistent and could be measured. APLP1 and 2 levels in the mutant GCPs were not distinguishable from those in wt controls (see also [42]). APLPs can partially substitute for APP function [50] so that some of this function persists in APP−/− neurons. Indeed, APP−/− mice are viable, but APP−/−APLP2−/− and complete APP family knock-out cause severe brain defects and perinatal lethality [20], [21], [51]. However, the absence of APP alone causes greatly reduced GC adhesion to laminin and, especially, to Abp, which suggests that the APLPs play a secondary role in GC adhesion. Yet, they likely account for most of the residual adhesion and outgrowth of APP−/− neurons on Abp in our experiments.

### APP is Present in GC Adhesions to Laminin

The colocalization of APP with Itga3b1 [12], [15], [16] in GCs on laminin was confirmed by biochemical studies. These show that adhesions of laminin-attached GCPs (enriched in Itgb1, CD81 and FAK) also contain a substantial amount of full-length APP, together with the APP-binding protein Dab1. Co-immunoprecipitation experiments confirmed in GCPs that APP interacts with Itga3b1 [11] as well as with CD81. However, our functional data indicate that APP-Itga3b1 complexes are not necessary for APP's adhesive function.

### Adhesion of GCs and Initial Outgrowth on Laminin are Dependent on APP Levels

On laminin, axonal GCs overexpressing APP were significantly enlarged as were their cumulative adhesive areas, and neurons with reduced, or lacking, APP expression had much smaller GCs and close adhesion areas. RICM is a well-established method for imaging close adhesions, and close-contact area correlates with cell adhesive force over a wide range (see Introduction). Therefore, the expression level of APP influences GC adhesion to laminin even though these GCs can interact with laminin via Itga3b1 and Itga7b1. This conclusion is supported by the finding that soluble Abp triggers about one half the GC collapse observed with both Abp and Ibp. Consequently APP plays an important role in GC adhesion. APP expression also affected neurite outgrowth on laminin (and on Abp). While the observed reduction of outgrowth of APP−/− neurons is consistent with findings in zebrafish [17] it varies from the results of Young-Pearse et al. [11], but the latter data were obtained on a polylysine-like matrix.

### Determining APP-Mediated Adhesion

Wt neurons grew on the mono-specific, laminin-derived peptide substrates as well as on the more physiological laminin. Their attachment and outgrowth on the synthetic matrices depended on the amount and sequence of peptide deposited, indicating biologically relevant specificity. This was supported further by the selectivity of peptide-induced GC collapse on laminin and on the mono-specific substrates. Finally, GCs formed typical close adhesions (as seen by RICM), with clustering of APP and Itga3 on Abp and Ibp matrices, respectively. However, we did not observe distinct, substrate-specific RICM patterns as reported by Gundersen's and Lemmon's laboratories [52], [53].

The peptide and eL1 matrices selectively engaged APP, Itgb1 family members or the L1 cell adhesion molecule in GC attachment. Wt axons grew equally well on the three synthetic substrates and laminin. This result differs from that reported by Lemmon et al. [24], who observed accelerated outgrowth on laminin compared to L1. The discrepancy most likely stems from differences in neuron type, matrix preparation (use of a nitrocellulose base vs. bare glass) and nature of the substrate (source of laminin; full-length L1 vs. recombinant eL1-Fc fusion protein). Of particular interest were close adhesions. We found that (i) close adhesion areas of wt GCs were similar on all three substrates, (ii) APP mutant neurons exhibited altered GC adhesions on Abp but not on the other matrices, and (iii) close adhesions of Itgb1− neurons were very small on Ibp but normal on the other matrices. The last result was of special interest because it showed that APP adhesion did not require Itgb1 participation (and vice versa, for APP−/− neurons on Ibp). Also important was the significant increase in close-adhesion area of hAPP+ GCs on Abp (vs. control substrates). Over the range from 0 to 1.9x wt level, close-adhesion area was about linearly dependent on APP protein dosage (Fig. 4G), assuming proportional surface expression. The APP-dependent increase in adhesion was confirmed by the enhanced resistance (relative to wt) of hAPP+ GCs to Abp-induced collapse (Fig. 4D, G). The disproportionate increase was most likely due to cooperative binding of clustered APP molecules [54], [55], [56].

**Figure 9:**
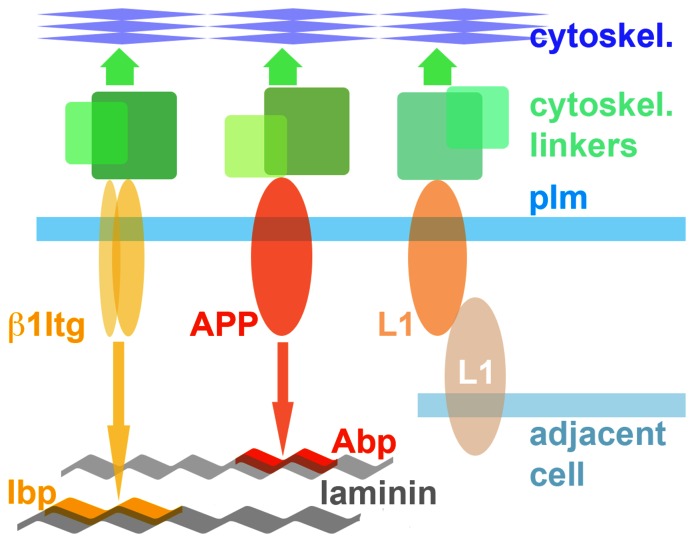
Schematic of GC-substrate interactions mediated by the three cell adhesion molecules of interest in this study. Itgb1 s and APP bind to distinct sequences on two different laminin subunits. Even though they can form complexes cell adhesion occurs independently, probably requiring distinct sets of cytoskeletal linker proteins.

Our studies establish that APP is an autonomously functioning GC cell adhesion molecule, and that APP-mediated adhesion by itself can sustain neurite outgrowth. Therefore, the observed APP-Itga3b1 complexes are not necessary for APP- or Itga3b1-mediated adhesion (see however [11]). Their functional role remains to be elucidated. Figure 9 illustrates schematically the interactions of the GC adhesion molecules Itgb1 s, APP and L1 with different growth substrates, laminin in the ECM or L1 on an adjacent cell surface. To be functional APP, like other adhesion molecules, must interact with the cytoskeleton via linker proteins. The APP-binding protein Apbb1 ( =  Fe65) is a strong candidate [16], but other known APP-binding proteins (Apba1  =  Mint1; Dab1) could be involved also. Cell adhesion and motility are interdependent [57] so that we anticipated APP-dependent changes in GC advance. APP−/− axons grew significantly more slowly on Abp compared to the other matrices or to wt. Interestingly, increasing APP dosage to 1.9x wt level (hAPP+) also reduced axonal outgrowth on Abp. The observed decreased motility below and above an adhesion optimum was consistent with other observations [58] and mathematical models that explain this biphasic relationship [7], [24], [59].

### APP Participates in GC Contact Guidance

Cells and GCs choose among potential substrates and advance directionally on gradients of differential adhesiveness (haptotactic behavior) indicating that adhesion molecules contribute to axonal pathfinding *in vivo*. We used substrate choice assays to test for APP participation in GC haptotaxis. The optimal method for quantitative analysis monitored neurites and GCs within a 5-µm border zone on either side of the interface of the paired matrices, where the GC's filopodia were likely to encounter the contrasting stripe. In the control experiments close to 50% of the outgrowth was observed on the juxtaposed side of the interface so that numbers in this range indicated stochastic growth across the stripes without substrate preference, and all three substrates fared essentially equally. Because of the very small numbers of mutant neurons that adhered to their non-permissive matrix (APP−/− on Abp; Itgb1− on Ibp) our results are limited to axons originating on their respective permissive matrices. In all permutations, neurites readily and randomly crossed onto eL1. Wt neurites grew randomly onto any one of the substrates (see also the comparison of physiologic substrates in [24], [60]). However, APP−/− neurites avoided a juxtaposed Abp matrix significantly, and Itgb1− neurites abstained from Ibp. Of special interest, hAPP+ axons averted crossover from Abp onto Ibp, in contrast to wt in the same experiment or to hAPP+ axons on an Ibp-eL1 pairing. Thus, hAPP+ neurites preferred the Abp matrix. Together, these data indicate that APP can influence contact guidance/haptotactic GC behavior.

### Conclusions

Comparisons of the adhesive functions of APP and two well-established adhesion molecules, the Itgb1 s and L1, demonstrate that APP-mediated spreading on the substrate, formation of close adhesions, and GC advance closely resemble those of GCs adhering via Itgb1 s or L1 only. Furthermore, our results indicate that APP significantly contributes to substrate choice in a manner analogous to that of Itgb1 s. Therefore, APP is an autonomously functioning adhesion molecule of the axonal GC that sustains outgrowth, participates in contact guidance mechanisms and, thus, may be involved in pathfinding. The importance of these results is further illustrated by the fact that APP expression levels significantly affect GC adhesion to laminin, even though laminin is a major adhesive substrate for Itgb1 s in the brain. These results raise the question of whether overexpression of APP in Down syndrome and perturbed APP proteolysis in familial Alzheimer disease affect wiring of the developing and, perhaps, plasticity of the mature nervous systems by a cell-autonomous mechanism.

## Materials and Methods

### Materials

Primary antibodies and their sources were: anti-N-terminal APP and anti-actin, Sigma-Aldrich Co. LLC (St. Louis, MO), used for immunofluorescence and blots in Figure 1; anti-C-terminal APP, Epitomics, Inc. (Burlingame, CA), used for all other western blots; anti-APLP1, anti-APLP2, anti-Dab1, anti-Gap43, and anti-Itgb1 (for immunofluorescence), Abcam PLC (Cambridge, MA); anti-Itga3 monoclonal antibody Ralph 3.1 (clone 6B3) from Developmental Studies Hybridoma Bank (University of Iowa, IA); anti-Itgb1 (for Western blots), anti-focal adhesion kinase (FAK), BD Biosciences Co. (San Jose, CA); anti-CD81, AbD Serotec, Morphosys Co. (Oxford, UK). Secondary antibodies: anti-rabbit IgG conjugated with Alexa Fluor 594 (red), anti-mouse IgG conjugated with Alexa Fluor 488 (green) or 555 (red), anti-rabbit IgG conjugated with Alexa Fluor 647 (Cy5; for Western blot), Cell Signaling Technology, Inc. (Boston, MA). Other fluorescent labels: Phalloidin conjugated with Alexa Fluor 488 or 555, Alexa Fluor 488-conjugated bovine serum albumin (BSA), Molecular Probes, Inc. (Eugene, OR).

Peptides were synthesized by GenicBio Co., Ltd. (Shenzhen, China) and checked mass-spectrometrically. The sequences of the APP-binding peptide (Abp) and its control were ARKQAASIKVAVS and KKSAVQARIVAS [6], [61]. The Itga3b1-binding peptide (Ibp) was PPFLMLFKSPKG [13] and its control MLFLPFPKPGSK.

The siRNAs targeting APP and Itgb1 and their control RNAs were obtained from Integrated DNA Technologies, Inc. (Coralville, IA). The following 3 sequences were used together.

APP-targeted siRNAs:

NM_019288 duplex 1 5′-AGAAUCCAACAUACAAGUUCUUUGA-3′

NM_019288 duplex 2 5′-GCCAAAGAGACAUGCAGUGAGAAGA-3′

NM_019288 duplex 3 5′-GUCAUAGCAACAGUGAUUGUCAUCA-3′

Itgb1− targeted siRNAs:

NM_010578 duplex 1 5′-GCUGGAGAACUAAUAGUUAAGAGAG-3′

NM_010578 duplex 2 5′-CCAAGUGACAUAGAGAAUCCCAGAG-3′

NM_010578 duplex 3 5′-AGCUCUCACUAGAUUGAAUGACACT-3.

The vector encoding wt APP was the generous gift of Dr. D.J. Selkoe (Harvard Institutes of Medicine, Boston, MA), [14]. Culture reagents and their sources were: Laminin, culture media, N2 and B27 supplements, Invitrogen Co. (Carlsbad, CA). All other reagents (purest grade available) were from Sigma-Aldrich Co. LLC (St. Louis, MO) or Thermo-Fisher Scientific Inc. (Waltham, MA).

### Animals

All animals in this study were maintained in an AAALAC-approved facility (Animal Welfare Assurance Number PHS A3269-01) and used in strict compliance with Protocol #B21711(01)1E (approved by the University of Colorado Denver's Animal Care and Use Committee), the U.S. Public Health Service's Policy on Humane Care and Use of Laboratory Animals and the *Guide for the Care and Use of Laboratory Animals.* Animals were sacrificed under deep terminal anesthesia and used for tissue collection only. Mouse breeders were obtained from The Jackson Laboratory, Bar Harbour, ME. The following mouse strains were used: Wild-type, C57BL/6J; APP−/−, B6.129S7-APP^tm1Dbo^/J [42]; hAPP+, B6.Cg-Tg(PDGFB-APP)5Lms [43]. Time-pregnant Sprague-Dawley rats were purchased from Harlan Sprague-Dawley Inc., Prattville, AL.

### GC Isolation

Mouse GC “particles” (GCPs) were isolated according to a protocol modified after [62], [63] that allows fractionation of individual newborn mouse brains. Brains were homogenized in 0.32 M sucrose containing 1 mM MgCl_2_, 2 mM TES buffer (2-[[1,3-dihydroxy-2-(hydroxymethyl)propan-2-yl]amino]ethanesulfonic acid), pH 7.3, and 2 µM aprotinin (all procedures at 4°C). The homogenate was spun for 7 min at 850 g_av_. The low-speed supernatant (LSS) was layered on a cushion of 0.83 M sucrose containing 1 mM MgCl_2_ and 2 mM TES and spun in an SW55Ti rotor (Beckman Coulter, Fullerton, CA) for 54 min at 286,794 g_av_. GCPs were collected from the 0.32/0.83 M sucrose interface, diluted with 0.32 M buffered sucrose and pelleted for 20 min at 76,000 g_av_. Rat GCPs were isolated according to [62], [63].

### Isolation of laminin-attached adhesion complexes

Fresh rat GCPs were gradually diluted with an equal volume of 2x modified Krebs buffer (0.05 M sucrose, 0.1 M NaCl, 5 mM KCl, 22 mM HEPES, 10 mM glucose, 1.2 mM NaH_2_PO_4_, 1.2 mM MgCl_2_, 2 mM CaCl_2_, pH 7.3) and incubated at 37°C for 10 min [64]. After incubation the GCPs were plated in laminin-coated 24-well culture plates, spun at 4,000 rpm for 15 min and incubated at 37°C for 30 min. After rinsing the plates twice with Tris-buffered saline (TBS), we added for extraction either 1% SDS (control) or 1% Brij98 in 5 mM MgCl_2_ and 5 µM CaCl_2_. The plates were incubated at 37°C for 20 min and then at 4°C for 40 min. Adhesion complexes were rinsed 2 times with TBS and subsequently lysed in 1% SDS. Chloroform/methanol (1:4)-precipitated proteins of all samples were resuspended in 5% SDS, and solubilized protein was analyzed by western blot. Because we were interested in comparing the distribution of specific proteins between the Brij98-soluble and Brij98-resistant fractions we loaded the same percentage of the sample yields in each lane (“equal fractional protein amounts”; non-extracted control samples were handled the same way).

### Co-Immunoprecipitation Experiments

For each immunoprecipitation, 30 µl protein G-agarose bead suspension (EMD Millipore Co., Billerica, MA) was incubated with 5 µg antibody diluted in PBS, for 3 hrs at 4°C with agitation. Fresh rat GCPs were pelleted and resuspended in ice-cold buffer (20 mM HEPES, 10 mM MgCl_2_, 5 µM CaCl_2_, 1 mM NaF, and protease inhibitors). After homogenization (Teflon/glass) the samples were spun at 22,000 g_av_ for 50 min. The pellet, composed of membranes and cytoskeletal elements, was solubilized in 2% Brij buffer (2% Brij 98, 5 mM MgCl_2_, 1 mM CaCl_2_ in TBS) by incubation at 37°C for 20 min, followed by 40 min at 4°C. The lysates were spun at 18,500 g_av_ for 15 min and the supernatant added to the washed, antibody-loaded beads for incubation overnight, at 4°C with constant agitation. Beads were collected by centrifugation, washed 3 times with PBS and resuspended in Laemmli buffer. The slurry was loaded on a polyacrylamide gel for electrophoresis and Western blot. Supernatant proteins were precipitated with chloroform/methanol (1∶4) and solubilized in SDS for Western blot analysis. For gel electrophoresis we loaded equal fractional sample amounts (load, supernatant, precipitate).

### Gel electrophoresis and Western blot

Samples were resolved by SDS-polyacrylamide gel electrophoresis and electrotransferred onto polyvinylidene fluoride (PVDF) membranes (Millipore Co., Billerica, MA). Blots were blocked with 5% non-fat milk and 0.1% Tween-20 in TBS for at least 1 hour, incubated with primary antibody for 1 hour (room temperature), washed with TBS-Tween-20, incubated with Cy5 fluorophore-conjugated secondary antibody for 1 hour and washed. Bound antibody was quantified in a laser fluorescence scanner (Typhoon 9400, GE Healthcare, Piscataway, NJ).

### Cell culture and transfection

Dissociated hippocampal pyramidal neurons were prepared from fetal E18 or newborn mouse brain and plated onto laminin-, peptide- or eL1-coated glass coverslips (Assistent Brand; Carolina Biological Supply Co., Burlington, NC). Peptide coating was achieved by incubating each 25-mm coverslip with 10 µg peptide in 285 µl PBS for 1 h at 37°C. eL1 was applied at 2.85 µg in 285 µl. For the experiments in Figure 4B a dilution series of the peptides was applied in the same manner. Thereafter, coverslips were quenched with 1% (wt/vol) BSA in PBS for 30 minutes. The cultures on laminin were maintained in Dulbecco's modified Eagle's medium plus N2 and B27 supplements, without serum [65] at 5% CO_2_/37°C. For culture on the synthetic matrices, however, we used only the N2 supplements, *minus putrescine*, in order to minimize non-specific adhesion. For some experiments dissociated wild-type neurons were transfected prior to plating with APP- or Itgb1− targeted siRNA (10 nM each of 3 sequences), or control siRNA, plus 1.5 µg pmaxGFP vector (Lonza Group Ltd, Basel, Switzerland) in 100 µl to label transfected cells. Other neurons were transfected with 3.2 µg APP vector plus 1.5 µg pmaxGFP in 100 µl. Transfection was by electroporation, using the optimized Amaxa Nucleofector (Lonza Group Ltd) protocol “mouse hippocampal neurons”.

### Collapse assays

Hippocampal neurons were plated onto laminin-, Abp-, Ibp- or eL1-coated glass coverslips. After 24 h in culture coverslips were mounted into an open chamber (Attofluor cell chamber, Molecular Probes/Invitrogen Co., Carlsbad, CA) with medium, layered over with inert mineral oil (embryo-tested, sterile-filtered; Sigma Aldrich Co. LLC, St Louis, MO) to maintain pH and avoid evaporation, and transferred to the microscope for live imaging under convective heating at 37°C. As phase contrast images were acquired the neurons were challenged by adding peptide at different concentrations to the medium. GC collapse was quantified by measuring (ImageJ software) the total area of the same live GC before and after treatment over a 10-minute period.

### Stripe assays

Matrices consisting of alternating, 55/45 µm-wide stripes of defined substrate were made essentially as described [45], [66], [67]. After placing the silicone “stamp” onto the coverslip the first peptide solution (75 µM in PBS) was infused and the assembly incubated for 1 h at 37°C. The channels were washed 2x to remove unbound peptide. In order to mark the first set of stripes, we incubated them with Alexa Fluor 488-BSA (150 nM) for 1 h at 37°C. After blocking with 1% (wt/vol) BSA in PBS for 30 minutes at 37°C we removed the stamp and added the second peptide (75 µM). The eL1 substrate was deposited in the same manner, at a concentration of 10 µg/ml. After incubation for 1 h at 37°C, we washed and blocked the stripes with 1% BSA in PBS for 30 minutes at 37°C and then substituted modified N2 medium. Dissociated hippocampal neurons were deposited on these matrices and allowed to grow for 1 day before fixation and processing for microscopy. We used the “border zone analysis to measure the relative GC preference for the juxtaposed matrices.

### Cell labeling and microscopy

Cells were fixed using slow infusion of 4% (wt/vol) formaldehyde in 0.1 M phosphate buffer, pH 7.4, with 120 mM glucose and 0.4 mM CaCl_2_ for a total of 30 min [68]. Cultures were rinsed with PBS containing 1 mM glycine, permeabilized for 2 minutes with 1% (vol/vol) Brij98 detergent in blocking buffer [PBS, with 1% (wt/vol) BSA] and placed in blocking buffer for 1 hour. Cultures were incubated with primary antibody for 1 hour, washed with blocking buffer (3x), labeled with Alexa Fluor 488- and/or 594-conjugated secondary antibodies (1 hour), and washed before embedding in Fluoromount-G (Southern Biotech, Birmingham, AL) reagent. Some samples were incubated with Alexa Fluor 488-phalloidin conjugate to label filamentous actin. These procedures were performed at room temperature. For the data in Figure 4E cells were processed as described except that fixation was with 2% formaldehyde for 10 min only, and GCs were imaged in PBS.

Images were acquired with a Zeiss Axiovert 200 M microscope with Zeiss optics (objectives: 40x Fluar 40x/1.3; Plan-Apo 63x/1.4; Alpha Plan-Apo 100x/1.46) and Cooke Sensicam camera, controlled by µManager software [69]. For live-cell imaging, cultures were placed in an open chamber with medium, layered over with inert mineral oil, and examined under convective heating (see above). GC adhesions were analyzed by RICM, which generates images based on the distance between the plasma membrane and the growth substratum [27]. Close adhesions were quantified using thresholding and area measurement (Metamorph software, Molecular Devices, LLC, Sunnyvale, CA) [70]. For total internal reflection fluorescence (TIRF) microscopy cells were examined with the 100x/1.46 objective, in combination with an argon ion laser-coupled TIRF illuminator (Zeiss).

### Statistics

Simple comparisons were examined for statistically significant differences by Student's two-sample t-test. However, the multiple comparisons of GC adhesive area, axonal outgrowth and GC substrate preference of 4 different neuron types (wt, mutants) on 3 different substrata required compensation for the increased risk of a Type I error. To this end we used a two-stage analysis. In stage 1 a two-way ANOVA tested the interaction between neuron type and substrate. If the F-test for interaction was significant then the nature of the interaction was evaluated in second-stage testing to determine whether specific neuron/matrix combinations experienced significantly different measurements in comparison to a control group consisting of combinations that were hypothesized (*a priori*) not to have altered measurements. The second stage comparisons used a multiple regression means model to identify the combinations with significant differences and to calculate estimates of the average differences with confidence intervals. There were twelve predictors in the means model representing the twelve different neuron/matrix combinations tested with the different outcome variables. The slope coefficient for each predictor in the model corresponded to the average value for each neuron/matrix combination from the data. This method provided a simple approach to performing multiple comparisons between the values of different neuron/matrix combinations. However, it was not desirable to test all possible comparisons because multiple combinations were not hypothesized to experience changed values. An *a-priori* control group needed to be established to which we could compare the groups with altered adhesion or growth rates. Because *wt* neurons should behave equally on all matrices, and because all neurons should behave equally on the eL1 matrix, these 6 groups were taken to represent adhesive area and growth rate under control conditions. The remaining six neuron/matrix combinations were each compared separately to the average growth in this combination control group (using an appropriate linear contrast in a cell-means model). In order to account for multiple comparisons, a Monte-Carlo simulation study was used to obtain a cutoff for the nominal p-value that maintains the desired experiment-wise type I-error rate at 0.05. The simulation study was conducted using normally-distributed data. The appropriateness of the resulting p-value cutoff was verified in a bootstrap study using the residuals from 12 group means to represent the null hypothesis. An analogous procedure was used for analyzing the stripe assay results. The control group for these assays is defined in Results.
